# Dissociative experiences alter resting state functional connectivity after childhood abuse

**DOI:** 10.1038/s41598-024-79023-9

**Published:** 2025-02-03

**Authors:** Claudius von Schröder, Richard O. Nkrumah, Traute Demirakca, Gabriele Ende, Christian Schmahl

**Affiliations:** 1 Department of Psychosomatic Medicine and Psychotherapy, Medical Faculty Mannheim, Central Institute of Mental Health, Heidelberg University, Mannheim, Germany; 2 Department of Neuroimaging, Medical Faculty Mannheim, Central Institute of Mental Health, Heidelberg University, Mannheim, Germany

**Keywords:** Attention, Diagnostic markers

## Abstract

Dissociative experiences commonly occur alongside adverse childhood experiences (ACE), yet research on their neurofunctional biomarkers has overlooked their unique association with dimensions of childhood abuse and neglect. We investigated interactions between dissociative experiences and childhood abuse, anticipating anti-correlations between the right-lateralized anterior middle frontal gyrus (raMFG) and the medial temporal lobe, as well as the temporal gyri. Examining resting-state functional connectivity in 91 participants with a history of ACE, we employed seed-to-voxel analyses seeding the raMFG. Multiple linear regression and post-hoc moderation/mediation models explored interactions and individual effects of dissociation and dimensions of ACE. The Dissociative Experiences Scale (DES) and Childhood Trauma Questionnaire (CTQ) quantified dissociation and dimensions of ACE. A DES by CTQ-A (childhood abuse) interaction predicted an anti-correlation between the raMFG and right hippocampus, moderated by CTQ-A. The CTQ revealed negative connectivity between the raMFG and right anterior cingulate cortex. CTQ-N (childhood neglect) indicated that both the right supplementary motor area and right insula related positively to the raMFG. Our findings underscore a distinct neural signature of childhood abuse-related dissociative experiences, potentially linked to dissociated memories.

## Introduction

Dissociative experiences are multifaceted psychological constructs prevalent across a wide spectrum of psychiatric conditions^1,2^. Frequently reported by individuals with a history of adverse childhood experiences (ACE), particularly abuse^3–9^, they can be thought of as contextually dependent coping strategies involving adaptations in emotion and memory regulation^8,10–14^ that offer protection when physical escape is not possible^15–19^. These adaptations primarily affect top-down inhibitory mechanisms of the prefrontal cortex and may thus counteract bottom-up fear-related signals stemming from the limbic system^2,12,13,18,20–23^. Despite substantial progress in examining the neurofunctional underpinnings of dissociation, prior research has largely focused on trauma-related dissociative experiences occurring within the context of post-traumatic stress disorder (PTSD)^13^, borderline personality disorder (BPD)^24^, and dissociative identity disorder (DID)^25^. Since existing studies have often overlooked the unique underlying dimensional influences of ACE, there remains a critical need to study the neurobiological mechanisms that connect dimensions of ACE to dissociative experiences and to explore how these factors interact.

Important work investigating the neurofunctional biomarkers of the trauma-related dissociative subtype of PTSD (PTSD+DS) has revealed distinctive functional connectivity patterns. Notably, these involve an overmodulation of limbic regions by disproportionate top-down prefrontal inhibition, distinguishing individuals with PTSD+DS from those with non-dissociative PTSD (PSTD-DS)^13,22,26–28^. This line of research builds on the Corticolimbic Disconnection Model proposed by Sierra and Berrios^21^, which focuses on depersonalization symptoms believed to involve “disconnections” within a corticolimbic brain circuit^21^.

More recent work has explored effective connectivity patterns involved in PTSD+DS-related corticolimbic disconnections^29–32^. In a study conducted by Nicholson and colleagues^32^, dynamic causal modeling analyses revealed a reduction in the modulation of the amygdala and periaqueductal gray (PAG) by the ventromedial prefrontal cortex (vmPFC) in individuals with PTSD-DS. This reduction indicated bottom-up processing, suggestive of diminished prefrontal inhibition. In contrast, individuals with PTSD+DS exuded increased modulation of the amygdala and PAG by the vmPFC, indicative of top-down processing and thus enhanced prefrontal inhibition. Interestingly, within the PTSD+DS group, the vmPFC demonstrated the strongest intrinsic inhibitory connections^32^.

Besides PTSD+DS, trauma-related dissociative experiences are highly prevalent in patients with BPD^1,33–35^. Neurofunctional biomarkers associated with dissociative experiences in BPD consistently show heightened prefrontal activity and decreased temporal activation during dissociation induction paradigms^36–39^. Building upon findings in PTSD+DS research^40–42^, Krause-Utz and colleagues^40^integrated a dissociation induction task with an Emotional Working Memory Task. They found that patients with current dissociation exhibited heightened activity in the inferior frontal gyrus^36^, consistent with related studies^38,39^. Furthermore, patients who completed the task following dissociation induction displayed decreased amygdala activity and diminished activity in the posterior cingulate, left cuneus, and lingual gyrus^36^.

Extending beyond PTSD+DS and BPD, dissociative experiences are most commonly observed in patients with DID, particularly in those with a history of ACE^1,20,43^. Similar to PTSD+DS, DID patients exhibit neurofunctional biomarkers indicative of disruptions in corticolimbic connectivity. For example, when comparing the ‘Neutral Identity State’ (NIS) to the ‘Trauma Identity State’ (TIS) in DID patients during exposure to personal trauma scripts, increased activation is observed in prefrontal regions, reflecting the emotion and memory overmodulation typically associated with PTSD+DS^10,20,22^. These areas include the right mid/anterior cingulate cortex, bilateral superior frontal gyrus, right middle frontal gyrus, and left medial frontal gyrus^10,20^. In contrast, compared to the NIS, the TIS in DID patients shows activation in subcortical regions such as the left amygdala, bilateral caudate, and left insula, while prefrontal and cingulate regions do not^5,6,10^. This pattern mirrors the neural signatures of classic PTSD-DS symptoms, characterized by hyperarousal and intrusive symptoms such as intrusive memories and flashbacks^22,44,45^.

Notably, activity in the bilateral parahippocampal gyri and posterior multimodal association areas, including the intraparietal sulcus, occipital cortex, and precuneus, has also been observed in DID patients’ NIS compared to their TIS during trauma scripts^10^. These activations likely reflect mechanisms specifically linked to dissociative amnesia^46–48^, facilitating emotional disengagement from trauma-related information^20,49^ and contributing to the active suppression of unwanted memories^50^. Structural neuroimaging studies support these observations, revealing significant reductions in hippocampal volume in DID patients, strongly associated with trauma-related dissociative experiences^5,6^. Adding to the evidence are specific volumetric reductions in the CA1 subfield of the hippocampus, which have been identified in DID patients with ACE-related dissociative experiences and suggested as a potential biomarker for dissociative amnesia^51^.

Additionally, the middle frontal gyrus, particularly regions within the anterior middle frontal gyrus such as the DLPFC, has been identified as a key prefrontal neurofunctional biomarker of dissociation in general^2^. Notably, the dorsolateral prefrontal cortex (DLPFC), especially the right DLPFC (rDLPFC), plays a crucial role in the modulation of memory and emotion^50,52–57^. The evidence reviewed here thus suggests that similar neural mechanisms involved in memory and emotion modulation are also implicated in dissociative experiences associated with PTSD^22^, BPD^24^, and DID^10^ via corticolimbic disconnections^13,21,22^. This implies that the predominant pattern of corticolimbic disconnections observed in dissociative experiences likely comprises top-down connectivity, exemplified by anti-correlations between regions within the right anterior middle frontal gyrus (raMFG; i.e., rDLPFC) involved in cognitive control, and subcortical regions within the medial temporal lobe (MTL; e.g., amygdala and hippocampus), as well as regions within the temporal gyri^2,10,13,23,24,51,58^.

Given the prominent relationship between ACE and dissociative experiences^4,5,7,9,43,59^, it is useful to briefly review related neurofunctional findings in the ACE literature. Intriguingly, when the influence of ACE is differentiated from that of PTSD-DS, neurofunctional biomarkers similar to those found in PTSD+DS, BPD, and DID-related dissociative experiences emerge. For example, after differentiating resting-state functional connectivity (RSFC) effects of ACE from those related to post-traumatic stress symptoms, Birn and colleagues^60^ discovered negative couplings between seed regions within the hippocampus and the vmPFC, dorsomedial prefrontal cortex, and DLPFC. Comparable negative relationships were observed between seed regions within the amygdala and the vmPFC, as well as the DLPFC^60^. Similarly, early life stress has been linked to changes in prefrontal RSFC, showing greater negative covariation between the DLPFC and the precuneus/inferior parietal lobule^61^. A related functional connectivity pattern was observed in women with a history of early life stress, showing negative connectivity between the DLPFC and amygdala^62^. Considering the overlap in findings, it seems plausible that the functional connectivity patterns observed in ACE, characterized by anti-correlations signifying corticolimbic disconnections, might serve as neurofunctional biomarkers for ACE-related dissociative experiences. These findings suggest the use of deliberate coping strategies involving emotion and memory regulation^12,28,41,63^. Such functional connectivity patterns may then be regarded as adaptations to ACE, likely involving differences in executive functioning and changes in autonomic, as well as interoceptive responses^64–68^.

Nonetheless, few studies have explicitly focused on functional connectivity alterations associated with ACE-related dissociative experiences. This complicates the interpretation of findings since both dissociative experiences and ACE are highly prevalent in psychiatric conditions such as PTSD, BPD, and DID. Moreover, there appears to be a unique association between traumatic stress and dissociative experiences when psychopathology is controlled for^69^. Thus, it remains to be determined to what extent neurofunctional biomarkers of trauma-related dissociative experiences are indeed due to dissociation, ACE, comorbid psychiatric conditions, or unique interactions between these factors^2^.

To understand the neurofunctional biomarkers specific to ACE-related dissociative experiences, we must also consider that ACE involve experiences of adversity that vary along dimensions of deprivation (neglect) and threat (abuse)^70^. Critically, McLaughlin and colleagues^70^ state that hitherto undifferentiated dimensions of early adversity significantly contribute to shaping neural development. An analysis of the neurobiological consequences of ACE-related dissociative experiences should then distinguish between these underlying dimensions, as previous failures to do so have likely led to inconsistent findings^70–72^.

Building on the findings reviewed above, we sought to investigate raMFG connectivity in response to dimensions of ACE-related dissociative experiences. Given the higher prevalence of dissociative experiences in childhood abuse compared to childhood neglect^4,7,9^, we hypothesized that the rDLPFC would exhibit anti-correlations with regions of the MTL (including the amygdala and hippocampus) and the temporal gyri in response to an interaction between dissociative experiences and childhood abuse. To test this hypothesis, we conducted seed-to-voxel functional connectivity analyses using the dorsal-ventral axes of the rDLPFC (Brodmann area 46, along with the dorsal and ventral Brodmann areas 9/46) as the seed region, which has been shown to integrate cognitive mechanisms related to executive control, emotional regulation, and self-referential processing^73^. We applied multiple linear regression models to assess the individual and interaction effects of our independent variables (IVs) on whole-brain raMFG connectivity. The IVs included the Dissociative Experiences Scale (DES) to quantify dissociative experiences and the Childhood Trauma Questionnaire (CTQ) to quantify childhood abuse and neglect, with additional clinical measures held constant to correct for their effects (see methods for more details).

## Results

Our analyses focused on whole-brain seed-to-voxel functional connectivity, using the raMFG as the seed region (see methods). We evaluated multiple linear regression models to examine the individual and interaction effects (the product of two IVs) of our psychometric measures quantifying dissociative experiences and ACE (see methods) on raMFG connectivity. Clusters that did not meet a voxel-discovery threshold of p-uncorrected < 0.001 and a cluster-level threshold of p-FDR (false discovery rate corrected p-value) < 0.05 were excluded^74^.

We began the seed-to-voxel analysis by evaluating the effects of dissociative experiences on raMFG connectivity through the individual impact of the total DES severity score. To isolate effects specific to total DES severity, we concurrently corrected for the influence of the CTQ, Brief Symptom Inventory (BSI), and Post-Traumatic Stress Disorder Checklist for DSM-5 (PCL-5) total severity scores, as well as age and sex. The analysis revealed no significant relationships.

**Fig. 1:**
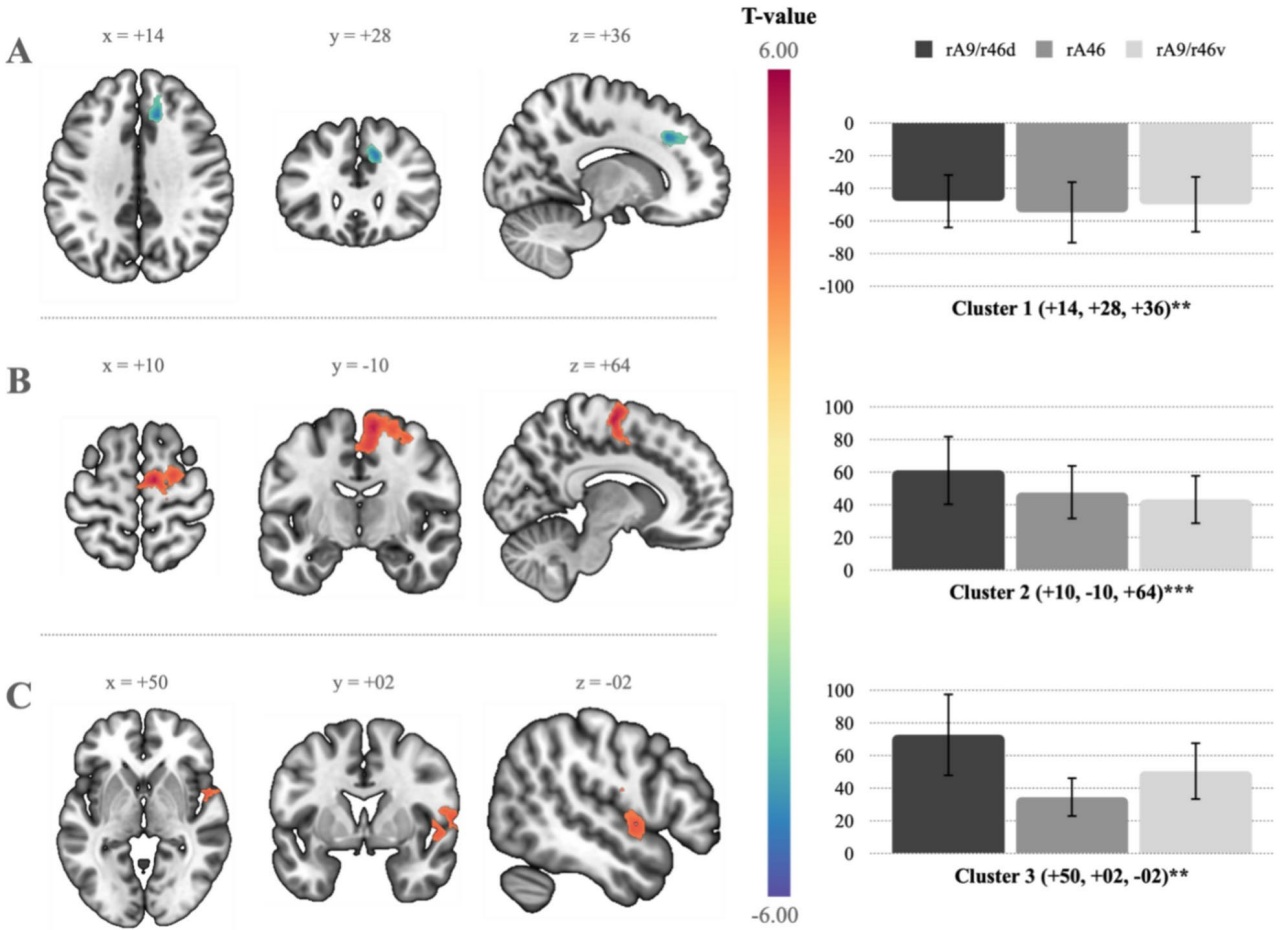
Seed-to-voxel analyses assessing whole-brain right anterior middle frontal gyrus (raMFG) connectivity in response to adverse childhood experiences (ACE). **A**) The CTQ (Childhood Trauma Questionnaire) predicted an anti-correlation between the raMFG and a cluster involving the rACC (right anterior cingulate cortex). **B**) CTQ-N (childhood neglect severity score) predicted positive correlations between the raMFG and a cluster involving the rSMA (right supplementary motor area), as well as **C**) a cluster involving the rINS (right insula). Warm t-values represent positive correlations, while cool t-values represent anti-correlations. Bar charts illustrate the percent signal change in BOLD (blood-oxygen-level-dependent) intensity for each cluster corresponding to the Brodmann areas within the raMFG seed region. Multiple comparisons were corrected with a voxel-discovery threshold of p-uncorrected < 0.001 and a cluster-level threshold of p-FDR < 0.05. Note: p-FDR < 0.05*, p-FDR < 0.01**, p-FDR < 0.001***. See Table 1 for MNI (Montreal Neurological Institute coordinate system) coordinates. rA9d/rA6d, right dorsal Brodmann area A9/A46. rA46, right Brodmann area 46. rA9v/rA6v, right ventral Brodmann area A9/A46. Images were rendered using MRIcroGL (https://www.nitrc.org/projects/mricrogl).

The effects of ACE were assessed by examining the individual impact of the total CTQ severity score on raMFG connectivity while concurrently correcting for the total DES, BSI, and PCL-5 severity scores, as well as age and sex. This analysis indicated a significant anti-correlation between the raMFG and a cluster within the right anterior cingulate cortex (rACC; see Table 1 and Fig. 1A).

Dimensions of ACE were examined by considering the total CTQ-A (CTQ-derived total childhood abuse severity score) and CTQ-N (CTQ-derived total childhood neglect severity score) severity scores individually. We first focused on raMFG connectivity associated exclusively with total CTQ-A severity by concurrently correcting for total CTQ-N severity, as well as the total DES, BSI, and PCL-5 severity scores, in addition to age and sex. No significant relationships were observed.

Conversely, we examined raMFG connectivity relative to total CTQ-N severity by correcting for CTQ-A, DES, BSI, and PCL-5 total severity scores, as well as age and sex. This yielded a significant positive correlation between the raMFG and a cluster involving the right supplementary motor area (rSMA; see Table 1 and Fig. 1B), as well as with a region involving the right insula (rINS; see Table 1 and Fig. 1C).

Interaction effects between dissociative experiences and dimensions of ACE were evaluated by first assessing the impact of the product of the total DES and CTQ severity scores (DES*CTQ) on raMFG connectivity. This was done while correcting for the total BSI and PCL-5 severity scores, as well as age and sex. This analysis did not provide any significant effects.

**Fig. 2:**
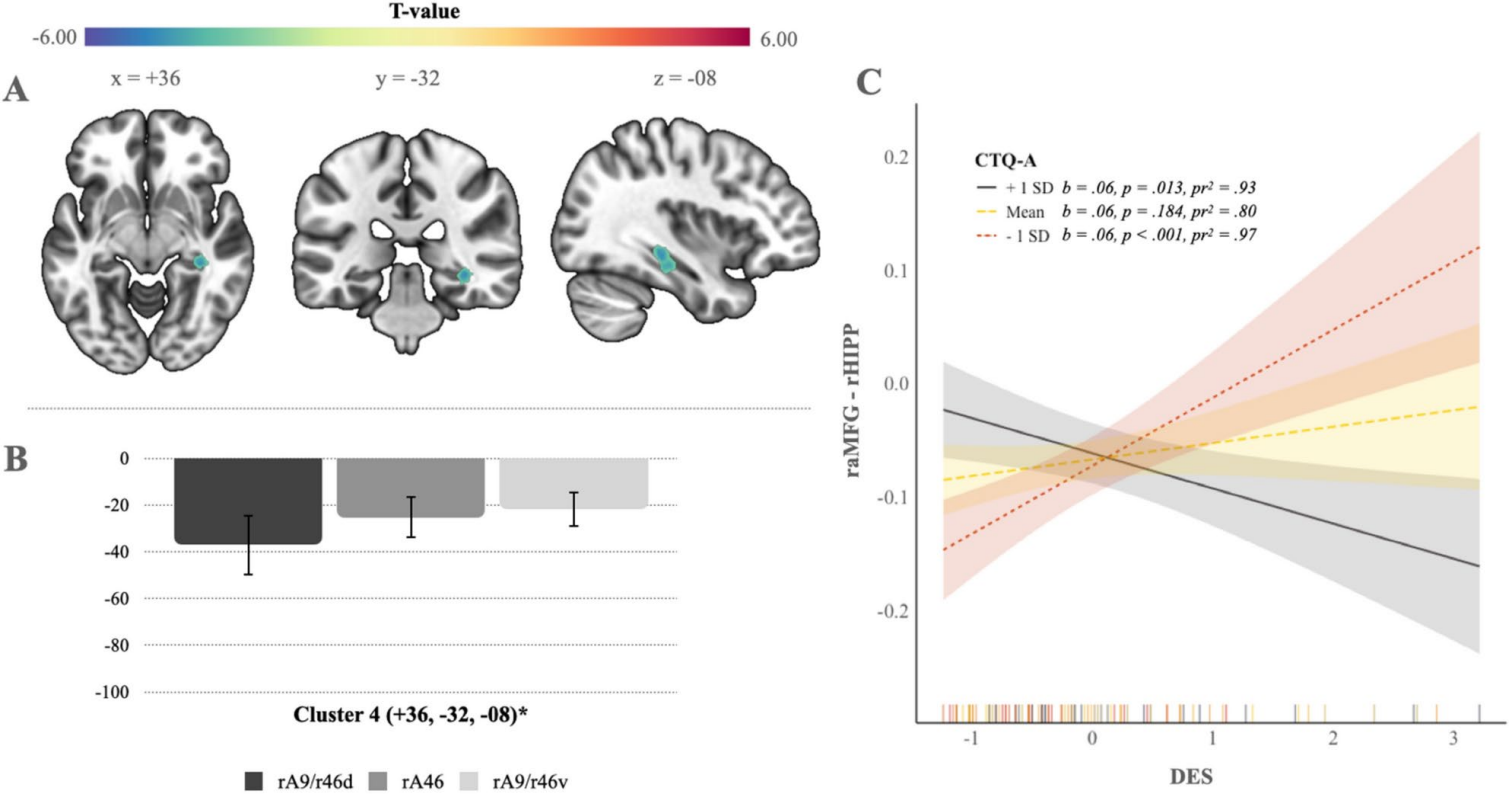
Seed-to-voxel analyses assessing whole-brain right anterior middle frontal gyrus (raMFG) connectivity in response to the interaction between dissociative experiences and childhood abuse. **A**) The interaction between the DES (Dissociative Experiences Scale) and CTQ-A (childhood abuse severity score) predicted an anti-correlation between the raMFG and a cluster defined by the rHIPP (right hippocampus). Multiple comparisons were corrected with a voxel-discovery threshold of p-uncorrected < 0.001 and a cluster-level threshold of p-FDR < 0.05. **B**) Percent signal change in BOLD (blood-oxygen-level-dependent) intensity for the rHIPP cluster corresponding to the Brodmann areas within the raMFG seed region. **C**) CTQ-A moderating the relationship between the DES and raMFG-rHIPP connectivity (*interaction effect* = − *.*04*, SE* = *.*01*,* 95% *CI* = [− *.*06*,* − *.*03]). For CTQ-A − 1SD, the DES related positively to raMFG-rHIPP connectivity (*b* = *.*06*, t*(82) = 3*.*92*, p* < *.*001, *pr*^*2*^ = .97), while CTQ-A + 1SD led to a negative relationship between the DES and raMFG-rHIPP connectivity (*b* = − *.*03*, t*(82) = − 2*.*53*, p* = *.*013, *pr*^*2*^ = .93). For mean CTQ-A, the DES was not significantly associated with raMFG-rHIPP connectivity (*b* = *.*01*, t*(82) = 1*.*34*, p* = *.*184, *pr*^*2*^ = .80). Note: p-FDR < 0.05*, p-FDR < 0.01**, p-FDR < 0.001***. See Table 1 for MNI (Montreal Neurological Institute coordinate system) coordinates. Images were rendered using MRIcroGL (https://www.nitrc.org/projects/mricrogl).

To examine our hypothesis, we then assessed interactions between the total DES and CTQ-A or CTQ-N severity scores. In each model, the opposing CTQ severity sub-score (i.e., either CTQ-A or CTQ-N) that was not included in the prior interaction model was held constant to correct for its effect. Age, sex, the BSI, and the PCL-5 total severity scores were also corrected for. While no significant interaction effects were observed between total DES and CTQ-N severity, the interaction between total DES and CTQ-A severity did predict a significant anti-correlation between the raMFG and a cluster involving the right hippocampus (rHIPP) (see Table 1 and Fig. 2A).

To further explore in how far the observed effect from the interaction between total DES and CTQ-A severity was conditional upon childhood abuse, we assessed a bootstrapped (10,000 samples) post-hoc moderation model. While the total model was significant (*F*(8*,*82) = 5*.*79*, p* < *.*001*, R*^2^ = *.*36), neither a main effect of total CTQ-A severity (*b* = *.*01*, SE* = *.*01*,*95% *CI* = [−*.*02*, .*04]) nor of total DES severity (*b* = *.*01*, SE* = *.*01*,*95% *CI* = [−*.*01*, .*04]) was observed. The addition of the interaction term (DES*CTQ-A), however, was significant (*interaction effect* = −*.*04*, SE* = *.*01*,* 95% *CI* = [−*.*06*,* −*.*03]; see Fig. 2C) and introduced a significant change to the model (*F*(1*,*82) = 30*.*61*, p* < *.*001*, R*^2^ *change* = *.*24), indicating a moderation effect (see Fig. 2C). Depending on total CTQ-A severity, the relationship between total DES severity and raMFG connectivity changed. For low CTQ-A scores, total DES severity related positively to raMFG-rHIPP connectivity (*b* = *.*06*, t*(82) = 3*.*92*, p* < *.*001, *pr*^*2*^ = .97), while high CTQ-A scores led to a negative relationship between total DES severity and raMFG-rHIPP connectivity (*b* = −*.*03*, t*(82) = −2*.*53*, p* = *.*013, *pr*^*2*^ = .93). At average CTQ-A scores, total DES severity was not significantly associated with raMFG-rHIPP connectivity (*b* = *.*01*, t*(82) = 1*.*34*, p* = *.*184, *pr*^*2*^ = .80).

Since the relationship between total DES severity and raMFG-rHIPP connectivity was conditional on total CTQ-A severity, we explored an additional bootstrapped (10,000 samples) post-hoc mediation model to investigate if total CTQ-A severity may, in fact, mediate this relationship. However, the mediation model yielded no significant effects. That is neither a total effect (*b* = −*.*00*, t*(82) = −*.*04*, p* = *.*969), direct effect (*b* = −*.*00*, t*(82) = −*.*037*, p* = *.*971), nor indirect effect (*indirect effect* = −*.*00*, SE* = *.*00*,* 95% *CI* [−*.*01*, .*00]) was observed.

**Table 1:** raMFG co-activation clusters.

Figure Index	Independent Variable	Brain Region	MNI Coordinates (x, y, z)	Cluster Size	T (p-FDR)
1A	CTQ	rACC	+ 14 + 28 + 36	224	−5.84 (.008)
1B	CTQ-N	rSMA	+ 10 −10 + 64	777	5.62 (.000)
1C	CTQ-N	rINS	+ 50 + 02 −02	300	4.69 (.001)
2A	DES x CTQ-A	rHIPP	+ 36 −32 −08	174	−5.53 (.023)

Note. Figure Index refers to the individual figures related to each raMFG co-activated cluster. CTQ, Childhood Trauma Questionnaire. CTQ-N, childhood neglect severity score. CTQ-A, childhood abuse severity score. DES, Dissociative Experiences Scale. rACC, right anterior cingulate cortex. rSMA, right supplementary motor area. rINS, right insula. rHIPP, right hippocampus.

## Discussion

Based on prior findings and the high prevalence of dissociative experiences occurring alongside childhood abuse^2,4–7,9,10,13,20,23,25,30,32,40,69^, we hypothesized that corticolimbic disconnections, signified by anti-correlations in RSFC between regions within the raMFG (i.e., rDLPFC) and MTL (i.e., amygdala and hippocampus), as well as the temporal gyri, would be influenced by interactions unique to dissociative experiences and childhood abuse. Before assessing our hypothesis, we explored the individual effects of each of our IVs. Surprisingly, we did not find any alterations in raMFG connectivity specific to dissociative experiences. However, we did discover that the total CTQ severity score predicted negative raMFG connectivity with a cluster involving the rACC (see Table 2 and Fig. 1A). This relates to findings demonstrating that ACE are associated with both functional and structural alterations of the ACC^71,72,75–80^. However, ACE-related RSFC studies of the ACC are sparse, particularly studies that corroborate our findings. Partially supporting our findings, van der Werff and colleagues^81^found that childhood emotional maltreatment was linked to reduced RSFC of the dorsal ACC (dACC), with the left dACC, precuneus, and angular cortex, but also with prefrontal areas, such as the mPFC, paracingulate gyrus, and the frontal pole^81^.

It is worth noting that the ACC is a core component of the Corticolimbic Disconnection Model of dissociation, where increased engagement of the PFC via the ACC is believed to reduce amygdala activity, resulting in an attenuation of affect^82,83^. Our raMFG-rACC anti-correlation, however, only relates to overall ACE severity, not to dissociative experiences. This suggests that raMFG-rACC anti-correlations may be indicative of ACE rather than ACE-related dissociative experiences.

Subsequent analyses explored the individual dimensional effects of childhood abuse and neglect on raMFG connectivity. CTQ-A-related raMFG connectivity did not yield any significant relationships. However, total CTQ-N severity predicted significant positive correlations between the raMFG and a cluster involving the rSMA (see Table 1 and Fig. 1B), as well as the rINS (see Table 1 and Fig. 1C). Both the rSMA and rINS are considered to be a part of the salience network (SN)^84,85^, indicating that childhood neglect may be related to increased raMFG-SN connectivity. Notably, a systematic review by McLaughlin and colleagues^71^found no SN alterations relative to childhood neglect. However, assuming the raMFG is involved in reorienting attention^86–89^, this connectivity pattern suggests that childhood neglect-related raMFG connectivity may be accompanied by shifts in attention that reflect SN-related processes such as interoception^90–92^.

After exploring the individual effects of our IVs, we examined our hypothesis by assessing interactions between dissociative experiences and dimensions of ACE. We focused our analysis on the raMFG due to its involvement in dissociation, as well as memory and emotion modulation. However, Fox and colleagues^86^and others^87,88,93–97^suggest that the raMFG may also facilitate a reorienting of attention to goal-relevant stimuli. Interestingly, Corbetta and colleagues^87^ argue that reorienting is not limited to attentional shifts between environmental inputs but may also facilitate a shift in attention focused on internal processes (involving the default network) to one focused on external stimuli and vice versa (depending on the goal). Thus, our observed raMFG-rHIPP anti-correlation in response to the interaction between the total DES and CTQ-A severity scores implies that childhood abuse-related dissociative experiences might involve a reorienting of attention from internal to external information. By suppressing hippocampal activity, this kind of attentional shift could help to avoid trauma-related self-generated thoughts indicative of depersonalization symptoms. The post-hoc moderation analysis, which indicated that total CTQ-A severity significantly moderated the relationship between total DES severity and raMFG-rHIPP connectivity (see Fig. 2C), further supports this view.

Indeed, our observed raMFG-rHIPP anti-correlation resembles findings from studies of enhanced memory control^55^. This is further paralleled by research demonstrating reduced hippocampal volume associated with dissociative amnesia specifically^51^and dissociation in general^2^. Intriguingly, Hulbert and Anderson^98^discovered that individuals with a history of ACE displayed greater memory control ability compared to a control group, though they did not consider dissociative experiences. During memory control tasks such as the Think/No-Think (TNT) task, participants are asked to stop memory retrieval by reorienting their attention to an external cue whenever a cue-related memory intrudes into their awareness^55,99^. Functional connectivity patterns related to enhanced memory control demonstrate the inhibitory effects on memory recall by top-down exertion of the raMFG (especially by the rDLPFC) over brain regions involved in memory reactivation^52,53,55,56,100–103^, resulting in a reduction of hippocampal activity^54,56,104^, particularly of the right hippocampus^125^, as well as volumetric reductions^105^.

When considering the specific instructions given during resting-state functional magnetic resonance imaging (rsfMRI) sessions^107^, similarities with instructions given during memory suppression tasks emerge^99^. During rsfMRI procedures, such as the one in our study, participants are explicitly instructed to clear their minds and think of nothing. They are also instructed to concentrate solely on a central fixation cross, which may help prevent potentially distracting thoughts^106,107^. Kawagoe and colleagues^106,107^investigated differences in rsfMRI instructions and found that being instructed to “think of nothing” during scanning sessions led to anti-correlations between prefrontal and default networks, including deactivation of the hippocampus. Further, this negative relationship became more pronounced as participants reported greater success in not thinking^106^.

Hence, in both situations, participants are instructed to “think of nothing” by reorienting their attention to an external cue when intruding thoughts occur, which weakens them. We speculate that the raMFG-rHIPP anti-correlation we observed is not only unique to childhood abuse-related dissociative experiences but also that dissociative experiences arising in chronic childhood abuse-related contexts may confer an enhanced memory control ability, allowing affected individuals to dissociate childhood abuse-related ACE from their memory. Future studies should address the cognitive strategies underlying these alterations in functional connectivity to confirm their involvement in memory control mechanisms related to dissociative amnesia.

While our findings provide valuable insights and important avenues for future research in clinical samples affected by pathological dissociation, they should be approached with some caution. Since we did not include a clinical sample, the extent to which these findings generalize to pathological dissociation remains uncertain. Future studies should consider including a clinical sample alongside an appropriate control group to determine whether the functional connectivity patterns we observed can be replicated within the context of dissociative disorders. Furthermore, in addition to self-report measures, using diagnostic interviews to assess the presence of pathological dissociation would enhance the reliability of the presence and severity of dissociative experiences.

In conclusion, we provide evidence that trauma-related dissociative experiences reported by individuals with a history of childhood abuse are unique^7^. While these experiences can cause dysfunction^108,109^, they may also offer protective benefits during and after traumatic events^110,111^. This protection might involve an alternative to physical escape by modulating attention to avoid intrusive trauma-related information, possibly indicating an enhanced memory control ability^66,98,112^. The interaction effects we observed between the total CTQ-A and DES severity scores on raMFG connectivity highlight the importance of investigating the unique combination of interacting psychosocial factors through which a dissociative experience may emerge^8,14,113^.

## Methods

### Participants

We enrolled 96 participants (79 female). The primary inclusion criterion for enrollment was a self-reported history of ACE within the first 18 years of life. Six participants were excluded from the final analysis. Two exhibited anomalies in their magnetic resonance images, likely attributed to movement artifacts during data acquisition. Another was excluded due to comprehension difficulties of several crucial questions during diagnostic interviews. Three were excluded due to incomplete clinical data. Consequently, the final data set used in our analyses consisted of 91 participants (see Table 2 for demographics).

Participants were recruited via flyers and advertisements. It is important to note that psychiatric conditions did not factor into our recruitment criteria. Instead, our study was designed to investigate brain alterations following ACE not specific to any psychiatric condition. Individuals below 18 and above 60 years of age were not eligible to participate. Further exclusion criteria included general magnetic resonance imaging (MRI) contraindications, such as the presence of metal implants or pregnancy, etc. A lifetime history of psychotic or bipolar-I disorders, as well as meeting criteria for a current substance use/abuse disorder, were also grounds for exclusion. So were psychotropic medications consumed within two weeks prior to participation. Exceptions were made for antidepressant medications such as selective serotonin reuptake inhibitors. Lastly, participants with a positive urine toxicology test were excluded from the study or re-invited later if abstinence could be maintained.

**Table 2:** Demographics and clinical measures.

	**ACE *(N* = *91)***
Sex, F / M	15 / 76
Age	31.36 (10.71)
DES	16.64 (13.15)
CTQ	94.77 (24.79)
CTQ-A	36.46 (13.06)
CTQ-N	27.35 (8.58)
PCL-5	28.17 (16.62)
BSI	00.89 (00.61)

Values include the mean (SD) or N. M, Male. F, Female. DES, Dissociative Experiences Scale. CTQ, Childhood Trauma Questionnaire. CTQ-A, childhood abuse severity score. CTQ-N, childhood neglect severity score. PCL-5, Post-Traumatic Stress Disorder Checklist for DSM-5. BSI, Brief Symptom Inventory.

### Psychometric measures

To assess dissociative experiences, we employed the German version of the DES (Fragebogen für Dissoziative Symptome)^114^. The German version includes an additional 16 items to capture pseudo-neurological conversion symptoms. However, we limited our use to the total DES severity score, which represents the mean of the same 28 items included in the original DES^115^. For a measure of overall ACE severity, we utilized the total sum score of the German version of the CTQ (i.e., the sum of all abuse [sexual, physical, and emotional] and neglect [emotional and physical] items)^116^. Additionally, we calculated separate sum scores for childhood abuse and neglect. For the childhood abuse severity score (CTQ-A), we summed items across sexual, physical, and emotional abuse. The same was done for emotional and physical neglect to obtain the childhood neglect severity score (CTQ-N). The German versions of the PCL-5^117^and BSI^118^ were used to obtain measures of post-traumatic stress symptoms and overall psychological distress. We incorporated the PCL-5 and BSI severity scores as covariates to correct for their effects, along with age and sex (see Table 2 for clinical characteristics).

### Procedure

The present study constituted a segment of an ongoing research initiative conducted as part of the Research Training Group 2350 (Graduiertenkolleg 2350), funded by the German Research Foundation (Deutsche Forschungsgemeinschaft). The overarching objective of this research endeavor was to explore the psychosocial and somatic implications arising from ACE, as previously detailed by Cackowski and Colleagues (2019)^119^. Our study received approval from the Ethical Board II of Heidelberg University, Germany, and was carried out in strict compliance with the principles set forth in the Declaration of Helsinki. All research procedures were explained to participants before their participation, after which they provided written informed consent.

Prior to MRI acquisition, participants completed questionnaires to quantify psychiatric symptom severities, as well as a history of ACE. Questionnaires were initially conducted in person at the Central Institute of Mental Health in Mannheim, Germany, but were subsequently completed online due to the local restrictions of the COVID-19 pandemic.

After completing a urine toxicology test, participants entered the MRI scanner to obtain structural and functional measurements. Earplugs and foam padding were applied to limit head movement and minimize scanner noise. During the blood-oxygen-level-dependent (BOLD) rsfMRI session, participants were instructed to remain awake and as motionless as possible. While keeping their eyes open, they were asked to direct their attention towards a central fixation cross and to think of nothing. Upon completion, participants received financial compensation for their time.

### MRI acquisition parameters

MRI data was acquired using a 3-T Prisma-fit Scanner (Siemens Medical Solutions, Erlangen, Germany) equipped with a 64-channel head coil. Initially, all participants underwent T1-weighted (T1w) anatomical imaging using a 3-D magnetization-prepared rapid-acquisition gradient echo (MPRAGE) sequence with the following parameters: Echo Time (TE) of 2.01 ms; Repetition Time (TR) of 2000 ms; Inversion Time (TI) of 900 ms; Flip Angle (FA) of 9°; Field of View (FOV) measuring 256 x 256 mm; 192 slices; and a voxel size of 1x1x1 mm^3^.

Subsequently, 400 BOLD rsfMRI images were collected employing a standard T2*-weighted echoplanar imaging (EPI) sequence with the following parameters: 36 slices in interleaved ascending order; TR of 1020 ms; TE of 30 ms; FA of 63°; FOV measuring 192x192 mm; matrix size of 64x64; voxel size of 3x3x3.75 mm3; MB factor of 2; in-plane acceleration factor of 2; the total duration was approximately 06:48 min.

### rsfMRI preprocessing

The rsfMRI data underwent preprocessing and noise reduction procedures following the recommended standard preprocessing pipeline for volume-based analyses using the CONN toolbox (http://www.nitrc. org/projects/conn) version 22a (Whitfield-Gabrieli&Nieto-Castanon,2012) in MATLAB 2021a (https://matlab.mathworks.com).

Specifically, the preprocessing pipeline included functional realignment and motion correction, slicetiming correction, spatial normalization to standard space (MNI-152), and spatial smoothing using a gaussian kernel with a full width at half-maximum (FWHM) of 8mm. Outlier scans were identified and removed using ART (http://www.nitrc.org/projects/artifact_detect).

To minimize cardiac and motion-related artifacts, the anatomical CompCor denoising method was applied to the functional data^120^. Artifacts identified using ART and the main effect of the scanning condition were also removed^120^. Moreover, a linear detrending step was incorporated into the preprocessing pipeline. Subsequently, the subject-specific denoised BOLD signal time-series underwent band-pass filtering, retaining signal frequencies within the 0.008 to 0.09 Hz range.

### Functional connectivity analyses

All RSFC analyses were conducted employing the CONN toolbox version 22a^121^. Our analyses focused on whole-brain seed-to-voxel connectivity, seeding the raMFG, which was sourced from the Brainnetome atlas^122^. The Brainnetome atlas offers a cross-validated, connectivity-based parcellation of 210 cortical and 36 subcortical brain regions^122^.

**Fig. 3:**
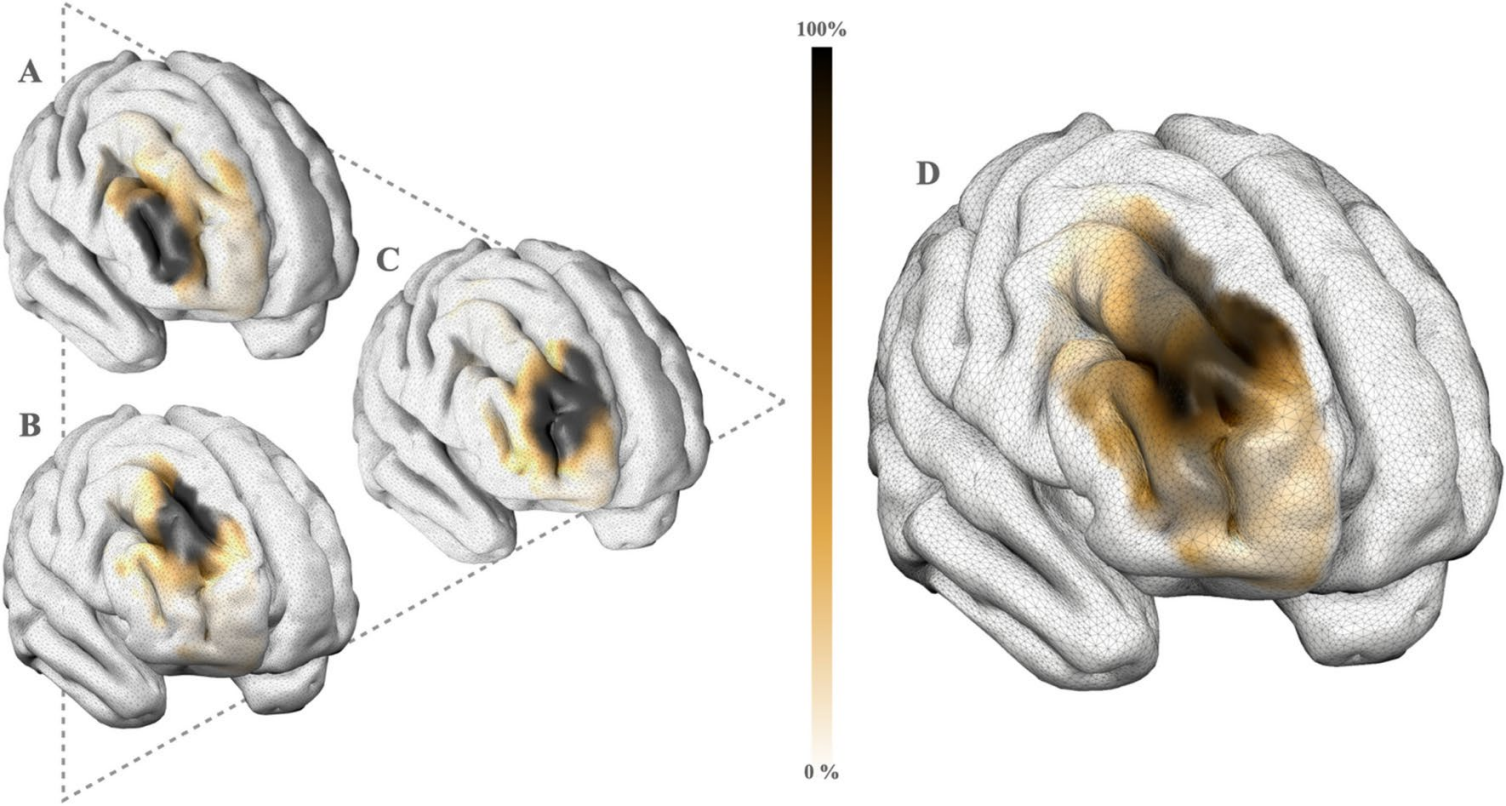
Right anterior middle frontal gyrus (raMFG) seed region. All functional connectivity analyses focused on whole-brain seed-to-voxel connectivity, using the raMFG as the seed region, derived from the Brainnetome atlas^122^. Specifically, the raMFG seed was obtained by averaging over the dorsal–ventral axes of the rDLPFC (right dorsolateral prefrontal cortex), which included Brodmann area 46 and both the dorsal and ventral Brodmann areas 9/46. A) Right ventral Brodmann area 9/46. B) Right dorsal Brodmann area 9/46. C) Right Brodmann area 46. D) Seed region: Average of A, B, and C. Darker colors represent seed regions with a higher probability of signal extraction, while lighter colors represent seed regions with a lower probability of signal extraction. Images were rendered using Surf Ice (https://www.nitrc.org/projects/surfice).

To conduct the seed-to-voxel analyses, the mean BOLD signal intensity time course for each subject was extracted by averaging over the anterior regions of the raMFG, specifically the dorsal-ventral axes of the rDLPFC including Brodmann area 46, as well as the dorsal and ventral Brodmann areas 9/46^73^ (see Fig. 3D). For each subject, the whole-brain FC maps were obtained by calculating the Fischer-transformed correlation coefficient between the time course of the seed and every voxel throughout the brain^123^. These connectivity maps were then entered into a second-level group analysis, wherein we evaluated several multiple linear regression models to examine individual effects and interactions (the product of two IVs) of our psychometric measures on raMFG connectivity. Prior to all analyses, all IVs and covariates were z-scored.

Clusters that did not meet a voxel-discovery threshold of p-uncorrected < 0.001 and a cluster-level threshold of p-FDR < 0.05 were excluded^74^. The MNI coordinates of peak clusters that were significantly related to the raMFG seed were entered into Neurosynth (https://www.neurosynth.org/locations/), where meta-analytical co-activation coefficients were assessed to aid in the interpretation of the anatomical locations of each co-activated cluster.

### Moderation and mediation analyses

To further explore the extent to which the observed effects in our interaction models might be conditional upon dissociative experiences, we examined post-hoc moderation (model-1) and mediation (model-4) models. These were performed using the PROCESS macro version 4.3^124^ in R. Standard errors and confidence intervals were bootstrapped with 10,000 bootstrap samples. To obtain simple slope-related effect sizes for the moderation analysis, T-values were converted to partial correlation coefficients (*pr*^*2*^) using the ‘t_to_r’ function from the ‘effectsize’ package^126^ in R. raMFG-rHIPP connectivity values from the seed-to-voxel RSFC analysis were exported from the CONN toolbox and entered as the dependent variable, while the total DES severity score was included as the independent variable. The total CTQ-A severity score served as the moderator or mediator variable, while the total CTQ-N severity score was concurrently adjusted for, along with age, sex, and the total BSI and PCL-5 severity scores. All continuous variables were mean centered for both the moderation and mediation models. Lastly, the moderator variable was assessed at the mean, as well as one standard deviation above and below the mean.

## Data Availability

Datasets created and/or analyzed during the present study will be made available at https://osf.io/xdw7h/.

## Abbreviations

ACC: Anterior cingulate cortex
ACE: Adverse childhood experiences
BOLD: Blood-oxygen-level-dependent
BPD: Borderline personality disorder
BSI: Brief Symptom Inventory
CTQ: Childhood Trauma Questionnaire
CTQ-A: CTQ-derived total childhood abuse severity score
CTQ-N: CTQ-derived total childhood neglect severity score
DES: Dissociative Experiences Scale
DID: Dissociative identity disorder
DLPFC: Dorsolateral prefrontal cortex
EPI: Echoplanar imaging
FA: Flip angle
FDR: False discovery rate
FOV: Field of view
FWHM: Full width at half-maximum
IV: Independent variable
MNI: Montreal Neurological Institute
MPRAGE: Magnetization-prepared rapid-acquisition gradient echo
MRI: Magnetic resonance imaging
MTL: Medial temporal lobe
NIS: Neutral Identity State
PAG: Periaqueductal gray
PCL-5: Post-Traumatic Stress Disorder Checklist for DSM-5
PTSD: Post-traumatic stress disorder
PTSD-DS: Non-dissociative PTSD
PTSD + DS: Dissociative subtype of PTSD
rACC: Right anterior cingulate cortex
raMFG: Right anterior middle frontal gyrus
rDLPFC: Right dorsolateral prefrontal cortex
rHIPP: Right hippocampus
rINS: Right insula
RSFC: Resting-state functional connectivity
rsfMRI: Resting-state functional magnetic resonance imaging
rSMA: Right supplementary motor area
SN: Salience network
TE: Echo Time
TI: Inversion time
TIS: Trauma Identity State
TNT: Think/No-Think task
TR: Repetition Time
vmPFC: Ventromedial prefrontal cortex

## References

[1] Lyssenko, L. et al. Dissociation in Psychiatric Disorders: A Meta-Analysis of Studies Using the Dissociative Experiences Scale. *Am. J. Psychiatry* **175**, 37–46. 10.1176/appi.ajp.2017.17010025 (2018). 28946763 10.1176/appi.ajp.2017.17010025

[2] Roydeva, M. I. & Reinders, A. A. Biomarkers of Pathological Dissociation: A Systematic Review. *Neurosci. & Biobehav. Rev.* **123**, 120–202. 10.1016/j.neubiorev.2020.11.019 (2021). 33271160 10.1016/j.neubiorev.2020.11.019

[3] Kate, M.-A. *Step 1: Instructions for clients MID-60 Multidimensional Inventory of Dissociation 60-item version* (2020).

[4] Vonderlin, R. et al. Dissociation in victims of childhood abuse or neglect: a meta-analytic review. *Psychol. Medicine* **48**, 2467–2476. 10.1017/S0033291718000740 (2018). 10.1017/S0033291718000740 29631646

[5] Chalavi, S. et al. Abnormal hippocampal morphology in dissociative identity disorder and posttraumatic stress disorder correlates with childhood trauma and dissociative symptoms. *Hum. Brain Mapp.* **36**, 1692–1704. 10.1002/hbm.22730 (2015). 25545784 10.1002/hbm.22730 PMC4400262

[6] Chalavi, S. et al. Similar cortical but not subcortical gray matter abnormalities in women with posttraumatic stress disorder with versus without dissociative identity disorder. *Psychiatry Res. Neuroimaging* **231**, 308–319. 10.1016/j.pscychresns.2015.01.014 (2015). 10.1016/j.pscychresns.2015.01.014 25670646

[7] Dorahy, M., Middleton, W., Seager, L., Williams, M. & Chambers, R. Child Abuse and Neglect in Complex Dissociative Disorder, Abuse-Related Chronic PTSD and Mixed Psychiatric Samples. *J. Trauma & Dissociation* 17, 10.1080/15299732.2015.1077916 (2015). 10.1080/15299732.2015.1077916 26275087

[8] Lynn, S. J. et al. Dissociation and Dissociative Disorders Reconsidered: Beyond Sociocognitive and Trauma Models Toward a Transtheoretical Framework. *Annu. Rev. Clin. Psychol.* **18**, 259–289. 10.1146/annurev-clinpsy-081219-102424 (2022). 35226824 10.1146/annurev-clinpsy-081219-102424

[9] Rafiq, S., Campodonico, C. & Varese, F. The relationship between childhood adversities and dissociation in severe mental illness: a meta-analytic review. *Acta Psychiatr. Scand.* 138, 509–525, 10.1111/acps.12969 (2018). _eprint: https://onlinelibrary.wiley.com/doi/pdf/10.1111/acps.12969. 10.1111/acps.12969 30338524

[10] Reinders, A. A. et al. Opposite brain emotion-regulation patterns in identity states of dissociative identity disorder: A PET study and neurobiological model. *Psychiatry Res. Neuroimaging* **223**, 236–243. 10.1016/j.pscychresns.2014.05.005 (2014). 10.1016/j.pscychresns.2014.05.005 24976633

[11] Vissia, E. M. et al. Dissociative identity state-dependent working memory in dissociative identity disorder: a controlled functional magnetic resonance imaging study. *BJPsych Open* **8**, e82. 10.1192/bjo.2022.22 (2022). 35403592 10.1192/bjo.2022.22 PMC9059616

[12] Fenster, R. J., Lebois, L. A. M., Ressler, K. J. & Suh, J. Brain circuit dysfunction in post-traumatic stress disorder: from mouse to man. *Nat. Rev. Neurosci.* 19, 535–551, 10.1038/s41583-018-0039-7 (2018). Number: 9 Publisher: Nature Publishing Group. 10.1038/s41583-018-0039-7 PMC6148363 30054570

[13] Lanius, R. A. et al. A Review of the Neurobiological Basis of Trauma-Related Dissociation and Its Relation to Cannabinoid- and Opioid-Mediated Stress Response: a Transdiagnostic. *Translational Approach. Curr. Psychiatry Reports* **20**, 118. 10.1007/s11920-018-0983-y (2018). 10.1007/s11920-018-0983-y 30402683

[14] Schimmenti, A. Dissociative experiences and dissociative minds: Exploring a nomological network of dissociative functioning. *J. Trauma & Dissociation* 17, 338–361, 10.1080/15299732.2015. 1108948 (2016). Publisher: Routledge _eprint: 10.1080/15299732.2015.1108948. 10.1080/15299732.2015.1108948 26507547

[15] Putnam, F. W. Child Development and Dissociation. *Child Adolesc. Psychiatr. Clin. North Am.* **5**, 285–302. 10.1016/S1056-4993(18)30367-5 (1996).

[16] Kluft, R. P. *Childhood Antecedents of Multiple Personality* (American Psychiatric Pub, 1985). Google-Books-ID: AplwBTXWr44C.

[17] Nijenhuis, E. R. S., Vanderlinden, J. & Spinhoven, P. Animal defensive reactions as a model for trauma-induced dissociative reactions. *J. Trauma. Stress.* 11, 243–260, 10.1023/A:1024447003022 (1998). _eprint: https://onlinelibrary.wiley.com/doi/pdf/10.1023/A%3A1024447003022. 10.1023/A:1024447003022 9565914

[18] Spiegel, D. Multiple personality as a post-traumatic stress disorder. *The Psychiatr. clinics North Am.* **7**, 101–110. 10.1016/s0193-953x(18)30783-4 (1984). 6718261

[19] Vermetten, E., Dorahy, M. J. & Spiegel, D. *Traumatic Dissociation: Neurobiology and Treatment* (American Psychiatric Pub, 2007). Google-Books-ID: F3GCJQYf9pIC.

[20] Reinders, A. A. T. S., Willemsen, A. T. M., Vos, H. P. J., Boer, J. A. d. & Nijenhuis, E. R. S. Fact or Factitious? A Psychobiological Study of Authentic and Simulated Dissociative Identity States. *PLOS ONE* 7, e39279, 10.1371/journal.pone.0039279 (2012). Publisher: Public Library of Science. 10.1371/journal.pone.0039279 PMC3387157 22768068

[21] Sierra, M. & Berrios, G. E. Depersonalization: neurobiological perspectives. *Biol. Psychiatry* **44**, 898–908. 10.1016/S0006-3223(98)00015-8 (1998). 9807645 10.1016/s0006-3223(98)00015-8

[22] Lanius, R. A. et al. Emotion modulation in PTSD: Clinical and neurobiological evidence for a dissociative subtype. *Am. J. Psychiatry* **167**, 640–647. 10.1176/appi.ajp.2009.09081168 (2010). 20360318 10.1176/appi.ajp.2009.09081168 PMC3226703

[23] Lebois, L. A. M. et al. Deconstructing dissociation: a triple network model of traumarelated dissociation and its subtypes. *Neuropsychopharmacology* **47**, 2261–2270. 10.1038/s41386-022-01468-1 (2022). 36202907 10.1038/s41386-022-01468-1 PMC9630268

[24] Krause-Utz, A., Frost, R., Winter, D. & Elzinga, B. M. Dissociation and Alterations in Brain Function and Structure: Implications for Borderline Personality Disorder. *Curr. Psychiatry Reports* 19, 10.1007/s11920-017-0757-y (2017). 10.1007/s11920-017-0757-y PMC5283511 28138924

[25] Reinders, A. A. T. S. & Veltman, D. J. Dissociative identity disorder: out of the shadows at last?. *The Br. J. Psychiatry* **219**, 413–414. 10.1192/bjp.2020.168 (2021). 33023686 10.1192/bjp.2020.168

[26] Brand, B. L., Lanius, R., Vermetten, E., Loewenstein, R. J. & Spiegel, D. Where Are We Going? An Update on Assessment, Treatment, and Neurobiological Research in Dissociative Disorders as We Move Toward the DSM-5. *J. Trauma & Dissociation* 13, 9–31, 10.1080/15299732.2011.620687 (2012). Publisher: Routledge _eprint: 10.1080/15299732.2011.620687. 10.1080/15299732.2011.620687 22211439

[27] Lanius, R. A., Bluhm, R., Lanius, U. & Pain, C. A review of neuroimaging studies in PTSD: Heterogeneity of response to symptom provocation. *J. Psychiatr. Res.* **40**, 709–729. 10.1016/j.jpsychires.2005.07.007 (2006). 16214172 10.1016/j.jpsychires.2005.07.007

[28] Lanius, R. A., Brand, B., Vermetten, E., Frewen, P. A. & Spiegel, D. THE DISSOCIATIVE SUBTYPE OF POSTTRAUMATIC STRESS DISORDER,. RATIONALE, CLINICAL AND NEUROBIOLOGICAL EVIDENCE, AND IMPLICATIONS: Dissociative Subtype of PTSD. Depress. Anxiety 29, 701–708. https://doi.org/10.1002/da.21889 (2012). 10.1002/da.21889 22431063

[29] Harricharan, S. *et al.* fMRI functional connectivity of the periaqueductal gray in PTSD and its dissociative subtype. *Brain Behav.* 6, e00579, 10.1002/brb3.579 (2016). _eprint: https://onlinelibrary.wiley.com/doi/pdf/10.1002/brb3.579. 10.1002/brb3.579 PMC5167004 28032002

[30] Nicholson, A. A. et al. The Dissociative Subtype of Posttraumatic Stress Disorder: Unique RestingState Functional Connectivity of Basolateral and Centromedial Amygdala Complexes. *Neuropsychopharmacology* **40**, 2317–2326. 10.1038/npp.2015.79 (2015). 25790021 10.1038/npp.2015.79 PMC4538346

[31] Nicholson, A. A. et al. Unique insula subregion resting-state functional connectivity with amygdala complexes in posttraumatic stress disorder and its dissociative subtype. *Psychiatry Res. Neuroimaging* **250**, 61–72. 10.1016/j.pscychresns.2016.02.002 (2016). 27042977 10.1016/j.pscychresns.2016.02.002

[32] Nicholson, A. A. *et al.* Dynamic causal modeling in PTSD and its dissociative subtype: Bottom–up versus top–down processing within fear and emotion regulation circuitry. *Hum. Brain Mapp.* 38, 5551–5561, 10.1002/hbm.23748 (2017). _eprint: https://onlinelibrary.wiley.com/doi/pdf/10.1002/hbm.23748. 10.1002/hbm.23748 PMC6866710 28836726

[33] Annegret Krause-Utz, Rachel Frost, Dorina Winter & M. Elzinga, B. Dissociation and Alterations in Brain Function and Structure: Implications for Borderline Personality Disorder. *Curr. Psychiatry Reports* 19, 10.1007/s11920-017-0757-y (2017). 10.1007/s11920-017-0757-y PMC5283511 28138924

[34] Knefel, M., Tran, U. S. & Lueger-Schuster, B. The association of posttraumatic stress disorder, complex posttraumatic stress disorder, and borderline personality disorder from a network analytical perspective. *J. Anxiety Disord.* **43**, 70–78. 10.1016/j.janxdis.2016.09.002 (2016). 27637074 10.1016/j.janxdis.2016.09.002

[35] Scalabrini, A., Cavicchioli, M., Fossati, A. & Maffei, C. The extent of dissociation in borderline personality disorder: A meta-analytic review. *J. Trauma & Dissociation* 18, 522–543, 10.1080/15299732.2016.1240738 (2017). Publisher: Routledge _eprint: 10.1080/15299732.2016.1240738. 10.1080/15299732.2016.1240738 27681284

[36] Krause-Utz, A. et al. Reduced amygdala reactivity and impaired working memory during dissociation in borderline personality disorder. *Eur. Arch. Psychiatry Clin. Neurosci.* **268**, 401–415. 10.1007/s00406-017-0806-x (2018). 28526931 10.1007/s00406-017-0806-x PMC5956011

[37] Krause-Utz, A. et al. Dissociation in Borderline Personality Disorder: Recent Experimental, Neurobiological Studies, and Implications for Future Research and Treatment. *Curr. Psychiatry Reports* **23**, 37. 10.1007/s11920-021-01246-8 (2021). 10.1007/s11920-021-01246-8 PMC8081699 33909198

[38] Ludäscher, P. *et al.* Pain sensitivity and neural processing during dissociative states in patients with borderline personality disorder with and without comorbid posttraumatic stress disorder: a pilot study. *J. Psychiatry Neurosci.* 35, 177–184, 10.1503/jpn.090022 (2010). Publisher: Journal of Psychiatry and Neuroscience Section: Research Paper. 10.1503/jpn.090022 PMC2861134 20420768

[39] Winter, D. et al. Dissociation in borderline personality disorder: Disturbed cognitive and emotional inhibition and its neural correlates. *Psychiatry Res. Neuroimaging* **233**, 339–351. 10.1016/j.pscychresns.2015.05.018 (2015). 10.1016/j.pscychresns.2015.05.018 26254542

[40] Krause-Utz, A. & Elzinga, B. Current Understanding of the Neural Mechanisms of Dissociation in Borderline Personality Disorder. *Curr. Behav. Neurosci. Reports* **5**, 113–123. 10.1007/s40473-018-0146-9 (2018). 10.1007/s40473-018-0146-9 PMC5857558 29577011

[41] Lanius, R. A. et al. Functional connectivity of dissociative responses in posttraumatic stress disorder: A functional magnetic resonance imaging investigation. *Biol. Psychiatry* **57**, 873–884. 10.1016/j.biopsych.2005.01.011 (2005). 15820708 10.1016/j.biopsych.2005.01.011

[42] Tursich, M. *et al.* Distinct intrinsic network connectivity patterns of post-traumatic stress disorder symptom clusters. *Acta Psychiatr. Scand.* 132, 29–38, 10.1111/acps.12387 (2015). _eprint: https://onlinelibrary.wiley.com/doi/pdf/10.1111/acps.12387. 10.1111/acps.12387 25572430

[43] Vissia, E. M. *et al.* Is it Trauma- or Fantasy-based? Comparing dissociative identity disorder, post-traumatic stress disorder, simulators, and controls. *Acta Psychiatr. Scand.* 134, 111–128, 10.1111/acps.12590 (2016). _eprint: https://onlinelibrary.wiley.com/doi/pdf/10.1111/acps.12590. 10.1111/acps.12590 27225185

[44] Felmingham, K. et al. Dissociative responses to conscious and non-conscious fear impact underlying brain function in post-traumatic stress disorder. *Psychol. Medicine* **38**, 1771–1780. 10.1017/S0033291708002742 (2008). 10.1017/S0033291708002742 18294420

[45] Hopper, J. W., Frewen, P. A., van der Kolk, B. A. & Lanius, R. A. Neural correlates of reexperiencing, avoidance, and dissociation in PTSD: symptom dimensions and emotion dysregulation in responses to script-driven trauma imagery. *J. Trauma. Stress.* **20**, 713–725. 10.1002/jts.20284 (2007). 17955540 10.1002/jts.20284

[46] Simeon, D. et al. Feeling unreal: a PET study of depersonalization disorder. *The Am. J. Psychiatry* **157**, 1782–1788. 10.1176/appi.ajp.157.11.1782 (2000). 11058475 10.1176/appi.ajp.157.11.1782

[47] Sar, V., Unal, S. N. & Ozturk, E. Frontal and occipital perfusion changes in dissociative identity disorder. *Psychiatry Res. Neuroimaging* **156**, 217–223. 10.1016/j.pscychresns.2006.12.017 (2007). 10.1016/j.pscychresns.2006.12.017 17961993

[48] Schlumpf, Y. R. *et al.* Dissociative part-dependent biopsychosocial reactions to backward masked angry and neutral faces: An fMRI study of dissociative identity disorder. *NeuroImage: Clin.* 3, 54–64, 10.1016/j.nicl.2013.07.002 (2013). 10.1016/j.nicl.2013.07.002 PMC3791283 24179849

[49] Reinders, A. a. T. S. *et al.* One brain, two selves. *NeuroImage* 20, 2119–2125, 10.1016/j. neuroimage.2003.08.021 (2003). 10.1016/j.neuroimage.2003.08.021 14683715

[50] Anderson, M. C. & Hanslmayr, S. Neural mechanisms of motivated forgetting. *Trends Cogn. Sci.* **18**, 279–292. 10.1016/j.tics.2014.03.002 (2014). 24747000 10.1016/j.tics.2014.03.002 PMC4045208

[51] Dimitrova, L. I. *et al.* A neurostructural biomarker of dissociative amnesia: a hippocampal study in dissociative identity disorder. *Psychol. Medicine* 1–9, 10.1017/S0033291721002154 (2021). 10.1017/S0033291721002154 PMC9975991 34165068

[52] Depue, B. E., Curran, T. & Banich, M. T. Prefrontal Regions Orchestrate Suppression of Emotional Memories via a Two-Phase Process. *Science* 317, 215–219, 10.1126/science.1139560 (2007). Publisher: American Association for the Advancement of Science. 10.1126/science.1139560 17626877

[53] Depue, B. E., Orr, J. M., Smolker, H. R., Naaz, F. & Banich, M. T. The Organization of Right Prefrontal Networks Reveals Common Mechanisms of Inhibitory Regulation Across Cognitive, Emotional, and Motor Processes. *Cereb. Cortex* **26**, 1634–1646. 10.1093/cercor/bhu324 (2016). 25601236 10.1093/cercor/bhu324 PMC4785949

[54] Gagnepain, P., Hulbert, J. & Anderson, M. C. Parallel Regulation of Memory and Emotion Supports the Suppression of Intrusive Memories. *The J. Neurosci.* **37**, 6423–6441. 10.1523/JNEUROSCI.2732-16 (2017). 28559378 10.1523/JNEUROSCI.2732-16.2017 PMC5511877

[55] Anderson, M. C. & Hulbert, J. C. Active Forgetting: Adaptation of Memory by Prefrontal Control. *Annu. Rev. Psychol.* 72, 1–36, 10.1146/annurev-psych-072720-094140 (2021). _eprint: 10.1146/annurev-psych-072720-094140. 10.1146/annurev-psych-072720-094140 32928060

[56] Benoit, R. G., Hulbert, J. C., Huddleston, E. & Anderson, M. C. Adaptive top-down suppression of hippocampal activity and the purging of intrusive memories from consciousness. *J. Cogn. Neurosci.* **27**, 96–111. 10.1162/jocn_a_00696 (2015). 25100219 10.1162/jocn_a_00696

[57] Anderson, M. C. & Floresco, S. B. Prefrontal-hippocampal interactions supporting the extinction of emotional memories: the retrieval stopping model. *Neuropsychopharmacology* 1–16, 10.1038/s41386-021-01131-1 (2021). Bandiera_abtest: a Cc_license_type: cc_by Cg_type: Nature Research Journals Primary_atype: Reviews Publisher: Nature Publishing Group Subject_term: Human behaviour;Neuroscience Subject_term_id: human-behaviour;neuroscience. 10.1038/s41386-021-01131-1 PMC8616908 34446831

[58] Lotfinia, S., Soorgi, Z., Mertens, Y. & Daniels, J. Structural and functional brain alterations in psychiatric patients with dissociative experiences: A systematic review of magnetic resonance imaging studies. *J. Psychiatr. Res.* **128**, 5–15. 10.1016/j.jpsychires.2020.05.006 (2020). 32480060 10.1016/j.jpsychires.2020.05.006

[59] Dalenberg, C. J. *et al.* Evaluation of the evidence for the trauma and fantasy models of dissociation. *Psychol. Bull.* 138, 550–588, 10.1037/a0027447 (2012). Place: US Publisher: American Psychological Association. 10.1037/a0027447 22409505

[60] Birn, R. M., Patriat, R., Phillips, M. L., Germain, A. & Herringa, R. J. Childhood Maltreatment and Combat Posttraumatic Stress Differentially Predict Fear-Related Fronto-Subcortical Connectivity. *Depress. Anxiety* 31, 880–892, 10.1002/da.22291 (2014). _eprint: https://onlinelibrary.wiley.com/doi/pdf/10.1002/da.22291. 10.1002/da.22291 PMC4205190 25132653

[61] Philip, N. S. et al. Early life stress impacts dorsolateral prefrontal cortex functional connectivity in healthy adults: Informing future studies of antidepressant treatments. *J. Psychiatr. Res.* **52**, 63–69. 10.1016/j.jpsychires.2014.01.014 (2014). 24513500 10.1016/j.jpsychires.2014.01.014 PMC3955403

[62] Kaiser, R. H. *et al.* Childhood stress, grown-up brain networks: corticolimbic correlates of threatrelated early life stress and adult stress response. *Psychol. Medicine* 48, 1157–1166, 10.1017/ S0033291717002628 (2018). Publisher: Cambridge University Press. 10.1017/S0033291717002628 PMC5867194 28942738

[63] Lanius, R. A. *et al.* The Nature of Traumatic Memories: A 4-T fMRI Functional Connectivity Analysis. *Am J Psychiatry* 9 (2004). 10.1176/appi.ajp.161.1.36 14702248

[64] Dell, P. F. A New Model of Dissociative Identity Disorder. *Psychiatr. Clin.* 29, 1–26, 10.1016/j.psc.2005.10.013 (2006). Publisher: Elsevier. 10.1016/j.psc.2005.10.013 16530584

[65] Fani, N. *et al.* Cognitive and neural facets of dissociation in a traumatized population. *Emotion* 19, 863–875, 10.1037/emo0000466 (2019). Place: US Publisher: American Psychological Association. 10.1037/emo0000466 PMC6382601 30124316

[66] Johnson, D. et al. Associations of Early-Life Threat and Deprivation With Executive Functioning in Childhood and Adolescence: A Systematic Review and Meta-analysis. *JAMA Pediatr.* **175**, e212511. 10.1001/jamapediatrics.2021.2511 (2021). 34309651 10.1001/jamapediatrics.2021.2511 PMC8314173

[67] McKinnon, M. C. et al. A review of the relation between dissociation, memory, executive functioning and social cognition in military members and civilians with neuropsychiatric conditions. *Neuropsychologia* **90**, 210–234. 10.1016/j.neuropsychologia.2016.07.017 (2016). 27444881 10.1016/j.neuropsychologia.2016.07.017

[68] Weniger, G. et al. Egocentric virtual maze learning in adult survivors of childhood abuse with dissociative disorders: Evidence from functional magnetic resonance imaging. *Psychiatry Res. Neuroimaging* **212**, 116–124. 10.1016/j.pscychresns.2012.11.004 (2013). 10.1016/j.pscychresns.2012.11.004 23522878

[69] Chiu, C.-D. et al. Cumulative traumatization associated with pathological dissociation in acute psychiatric inpatients. *Psychiatry Res.* **230**, 406–412. 10.1016/j.psychres.2015.09.028 (2015). 26454403 10.1016/j.psychres.2015.09.028

[70] McLaughlin, K. A., Sheridan, M. A. & Lambert, H. K. Childhood adversity and neural development: Deprivation and threat as distinct dimensions of early experience. *Neurosci. & Biobehav. Rev.* **47**, 578–591. 10.1016/j.neubiorev.2014.10.012 (2014). 25454359 10.1016/j.neubiorev.2014.10.012 PMC4308474

[71] McLaughlin, K. A., Weissman, D. & Bitrán, D. Childhood Adversity and Neural Development: A Systematic Review. *Annu. Rev. Dev. Psychol.* 1, 277–312, 10.1146/ annurev-devpsych-121318–084950 (2019). _eprint: 10.1146/annurev-devpsych121318-084950. 10.1146/annurev-devpsych-121318-084950 PMC7243625 32455344

[72] Nemeroff, C. Paradise Lost: The Neurobiological and Clinical Consequences of Child Abuse and Neglect. *Neuron* **89**, 892–909. 10.1016/j.neuron.2016.01.019 (2016). 26938439 10.1016/j.neuron.2016.01.019

[73] Jung, J., Lambon Ralph, M. A. & Jackson, R. L. Subregions of DLPFC Display Graded yet Distinct Structural and Functional Connectivity. *The J. Neurosci.* 42, 3241–3252, 10.1523/JNEUROSCI.1216–21.2022 (2022). 10.1523/JNEUROSCI.1216-21.2022 PMC8994544 35232759

[74] Worsley, K. J. *et al.* A unified statistical approach for determining significant signals in images of cerebral activation. *Hum. Brain Mapp.* 4, 58–73. https://doi.org/10.1002/(SICI)1097-0193(1996)4:1<58::AID-HBM4>3.0.CO;2-O (1996). _eprint: 1. https://onlinelibrary.wiley.com/doi/pdf/10.1002/%28SICI%2910970193%281996%294%3A1%3C58%3A%3AAID-HBM4%3E3.0.CO%3B2-O. 10.1002/(SICI)1097-0193(1996)4:1<58::AID-HBM4>3.0.CO;2-O 20408186

[75] Hart, H. & Rubia, K. Neuroimaging of child abuse: a critical review. *Front. Hum. Neurosci.* 6 (2012). 10.3389/fnhum.2012.00052 PMC3307045 22457645

[76] Herzog, J. I. & Schmahl, C. Adverse Childhood Experiences and the Consequences on Neurobiological, Psychosocial, and Somatic Conditions Across the Lifespan. *Front. Psychiatry* **9**, 420. 10.3389/fpsyt.2018.00420 (2018). 30233435 10.3389/fpsyt.2018.00420 PMC6131660

[77] Teicher, M. H. & Samson, J. A. Annual Research Review: Enduring neurobiological effects of childhood abuse and neglect. *J. Child Psychol. Psychiatry* **57**, 241–266. 10.1111/jcpp.12507 (2016). 26831814 10.1111/jcpp.12507 PMC4760853

[78] Teicher, M. H., Samson, J. A., Anderson, C. M. & Ohashi, K. The effects of childhood maltreatment on brain structure, function and connectivity. *Nat. Rev. Neurosci.* **17**, 652–666. 10.1038/nrn.2016.111 (2016). 27640984 10.1038/nrn.2016.111

[79] Teicher, M. H. & Samson, J. A. Childhood maltreatment and psychopathology: A case for ecophenotypic variants as clinically and neurobiologically distinct subtypes. *Am. J. Psychiatry* **170**, 1114–1133. 10.1176/appi.ajp.2013.12070957 (2013). 23982148 10.1176/appi.ajp.2013.12070957 PMC3928064

[80] Reinders, A. a. T. S. *et al.* Neurodevelopmental origins of abnormal cortical morphology in dissociative identity disorder. *Acta Psychiatr. Scand.* 137, 157–170, 10.1111/acps.12839 (2018). _eprint: https://onlinelibrary.wiley.com/doi/pdf/10.1111/acps.12839. 10.1111/acps.12839 29282709

[81] van der Werff, S. J. A. et al. Resting-state functional connectivity in adults with childhood emotional maltreatment. *Psychol. Medicine* **43**, 1825–1836. 10.1017/S0033291712002942 (2013). 10.1017/S0033291712002942 23254143

[82] Sierra, M. et al. Autonomic Response in Depersonalization Disorder. *Arch. Gen. Psychiatry* **59**, 833. 10.1001/archpsyc.59.9.833 (2002). 12215083 10.1001/archpsyc.59.9.833

[83] Sierra, M., Senior, C., Phillips, M. L. & David, A. S. Autonomic response in the perception of disgust and happiness in depersonalization disorder. *Psychiatry Res.* **145**, 225–231. 10.1016/j.psychres.2005.05.022 (2006). 17074399 10.1016/j.psychres.2005.05.022

[84] Seeley, W. W. *et al.* Dissociable intrinsic connectivity networks for salience processing and executive control. *The J. Neurosci. The Off. J. Soc. for Neurosci.* 27, 2349–2356, 10.1523/JNEUROSCI. 5587–06.2007 (2007). 10.1523/JNEUROSCI.5587-06.2007 PMC2680293 17329432

[85] Uddin, L. Q. Salience processing and insular cortical function and dysfunction. *Nat. Rev. Neurosci.* 16, 55–61, 10.1038/nrn3857 (2015). Number: 1 Publisher: Nature Publishing Group. 10.1038/nrn3857 25406711

[86] Fox, M. D., Corbetta, M., Snyder, A. Z., Vincent, J. L. & Raichle, M. E. Spontaneous neuronal activity distinguishes human dorsal and ventral attention systems. *Proc. Natl. Acad. Sci.* 103, 10046– 10051, 10.1073/pnas.0604187103 (2006). Publisher: Proceedings of the National Academy of Sciences. 10.1073/pnas.0604187103 PMC1480402 16788060

[87] Corbetta, M., Patel, G. & Shulman, G. L. The Reorienting System of the Human Brain: From Environment to Theory of Mind. *Neuron* 58, 306–324, 10.1016/j.neuron.2008.04.017 (2008). Publisher: Elsevier. 10.1016/j.neuron.2008.04.017 PMC2441869 18466742

[88] Japee, S., Holiday, K., Satyshur, M. D., Mukai, I. & Ungerleider, L. G. A role of right middle frontal gyrus in reorienting of attention: a case study. *Front. Syst. Neurosci.* 0, 10.3389/fnsys.2015. 00023 (2015). Publisher: Frontiers. 10.3389/fnsys.2015.00023 PMC4347607 25784862

[89] Corbetta, M. & Shulman, G. L. Control of goal-directed and stimulus-driven attention in the brain. *Nat. Rev. Neurosci.* 3, 201–215, 10.1038/nrn755 (2002). Number: 3 Publisher: Nature Publishing Group. 10.1038/nrn755 11994752

[90] Menon, V. & Uddin, L. Q. Saliency, switching, attention and control: a network model of insula function. *Brain structure & function* **214**, 655–667. 10.1007/s00429-010-0262-0 (2010). 20512370 10.1007/s00429-010-0262-0 PMC2899886

[91] Uddin, L. Q., Yeo, B. T. & Spreng, R. N. Towards a universal taxonomy of macro-scale functional human brain networks. *Brain topography* **32**, 926–942. 10.1007/s10548-019-00744-6 (2019). 31707621 10.1007/s10548-019-00744-6 PMC7325607

[92] Menon, V. Large-scale brain networks and psychopathology: a unifying triple network model. *Trends Cogn. Sci.* **15**, 483–506. 10.1016/j.tics.2011.08.003 (2011). 21908230 10.1016/j.tics.2011.08.003

[93] De Schotten, M. T. et al. Direct Evidence for a Parietal-Frontal Pathway Subserving Spatial Awareness in Humans. *Science* **309**, 2226–2228. 10.1126/science.1116251 (2005). 16195465 10.1126/science.1116251

[94] Suo, X. et al. Anatomical and functional coupling between the dorsal and ventral attention networks. *NeuroImage* **232**, 117868. 10.1016/j.neuroimage.2021.117868 (2021). 33647500 10.1016/j.neuroimage.2021.117868

[95] Vossel, S., Geng, J. J. & Fink, G. R. Dorsal and Ventral Attention Systems: Distinct Neural Circuits but Collaborative Roles. *The Neurosci.* **20**, 150–159. 10.1177/1073858413494269 (2014). 10.1177/1073858413494269 PMC4107817 23835449

[96] He, B. J. et al. Breakdown of Functional Connectivity in Frontoparietal Networks Underlies Behavioral Deficits in Spatial Neglect. *Neuron* **53**, 905–918. 10.1016/j.neuron.2007.02.013 (2007). 17359924 10.1016/j.neuron.2007.02.013

[97] Corbetta, M. & Shulman, G. L. Spatial Neglect and Attention Networks. *Annu, Rev. Neurosci.* 34, 569–599, 10.1146/annurev-neuro-061010-113731 (2011). _eprint: 10.1146/annurev-neuro-061010-113731. 10.1146/annurev-neuro-061010-113731 PMC3790661 21692662

[98] Hulbert, J. C. & Anderson, M. C. What doesn’t kill you makes you stronger: Psychological trauma and its relationship to enhanced memory control. *J. Exp. Psychol. Gen.* **147**, 1931–1949. 10.1037/xge0000461 (2018). 30024184 10.1037/xge0000461 PMC6277128

[99] Nardo, D. & Anderson, M. Everything You Ever Wanted to Know About the Think/No-Think task, But Forgot to Ask. preprint, PsyArXiv (2023). 10.31234/osf.io/t3dn4. 10.3758/s13428-024-02349-9 PMC11133138 38379115

[100] Benoit, R. & Anderson, M. Opposing Mechanisms Support the Voluntary Forgetting of Unwanted Memories. *Neuron* **76**, 450–460. 10.1016/j.neuron.2012.07.025 (2012). 23083745 10.1016/j.neuron.2012.07.025 PMC3480638

[101] Gagnepain, P., Henson, R. N. & Anderson, M. C. Suppressing unwanted memories reduces their unconscious influence via targeted cortical inhibition. *Proc. Natl. Acad. Sci.* 111, 10.1073/pnas.1311468111 (2014). 10.1073/pnas.1311468111 PMC3977236 24639546

[102] Mary, A. *et al.* Resilience after trauma: The role of memory suppression. *Science* 367, 10.1126/science.aay8477 (2020). Publisher: American Association for the Advancement of Science Section: Research Article. 10.1126/science.aay8477 32054733

[103] Schmitz, T. W., Correia, M. M., Ferreira, C. S., Prescot, A. P. & Anderson, M. C. Hippocampal GABA enables inhibitory control over unwanted thoughts. *Nat. Commun.* 8, 1311, 10.1038/ s41467–017–00956-z (2017). Number: 1 Publisher: Nature Publishing Group. 10.1038/s41467-017-00956-z PMC5670182 29101315

[104] Levy, B. J. & Anderson, M. C. Purging of Memories from Conscious Awareness Tracked in the Human Brain. *The J. Neurosci.* **32**, 16785–16794. 10.1523/JNEUROSCI.2640-12.2012 (2012). 23175832 10.1523/JNEUROSCI.2640-12.2012 PMC3544307

[105] Depue, B. & Banich, M. Increased inhibition and enhancement of memory retrieval are associated with reduced hippocampal volume. *Hippocampus* 22, 651–655, 10.1002/hipo.20952 (2012). _eprint: https://onlinelibrary.wiley.com/doi/pdf/10.1002/hipo.20952. 10.1002/hipo.20952 PMC3191317 21656873

[106] Kawagoe, T., Onoda, K. & Yamaguchi, S. Different pre-scanning instructions induce distinct psychological and resting brain states during functional magnetic resonance imaging. *Eur. J. Neurosci.* 47, 77–82, 10.1111/ejn.13787 (2018). _eprint: https://onlinelibrary.wiley.com/doi/pdf/10.1111/ejn.13787. 10.1111/ejn.13787 29205574

[107] Kawagoe, T., Onoda, K. & Yamaguchi, S. The neural correlates of “mind blanking”: When the mind goes away. *Hum. Brain Mapp.* 40, 4934–4940, 10.1002/hbm.24748 (2019). _eprint: https://onlinelibrary.wiley.com/doi/pdf/10.1002/hbm.24748. 10.1002/hbm.24748 PMC6865483 31389642

[108] Franzke, I., Wabnitz, P. & Catani, C. Dissociation as a Mediator of the Relationship Between Childhood Trauma and Nonsuicidal Self-Injury in Females: A Path Analytic Approach. *J. Trauma & Dissociation* 16, 286–302, 10.1080/15299732.2015.989646 (2015). Publisher: Routledge _eprint: 10.1080/15299732.2015.989646. 10.1080/15299732.2015.989646 25761222

[109] Kratzer, L. et al. Mindfulness and pathological dissociation fully mediate the association of childhood abuse and PTSD symptomatology. *Eur. J. Trauma & Dissociation* **2**, 5–10. 10.1016/j.ejtd.2017.06.004 (2018).

[110] Békés, V. The way we are: how states of mind influence our identities, personality and potential for change. *J. Trauma & Dissociation* 21, 144–145, 10.1080/15299732.2019.1666451 (2020). Publisher: Routledge _eprint: 10.1080/15299732.2019.1666451.

[111] Griffin, M. G., Resick, P. A. & Mechanic, M. B. Objective Assessment of Peritraumatic Dissociation: Psychophysiological Indicators. *The Am. journal psychiatry* **154**, 1081–1088 (1997). 9247393 10.1176/ajp.154.8.1081 PMC2958429

[112] Dell, P. F. & O’Neil, J. A. *Dissociation and the Dissociative Disorders: DSM-V and Beyond* (Routledge, 2010). Google-Books-ID: aEuTAgAAQBAJ.

[113] McNally, R. J.Network Analysis of Psychopathology: Controversies and Challenges. *Annu. Rev. Clin. Psychol.* 17, 31–53, 10.1146/annurev-clinpsy-081219-092850 (2021). _eprint: 10.1146/annurev-clinpsy-081219-092850. 10.1146/annurev-clinpsy-081219-092850 33228401

[114] Freyberger, H. J. *et al.* Fragebogen zu dissoziativen Symptomen (FDS). Deutsche Adaptation, Reliabilität und Validität der amerikanischen Dissociative Experience Scale (DES). [The Fragebogen (Questionnaire) zu dissoziativen Symptomen (FDS): German adaptation, reliability, and validity of the American Dissociative Experience Scale (DES).]. *PPmP: Psychother. Psychosom. Medizinische Psychol.* 48, 223–229 (1998). Place: Germany Publisher: Georg Thieme Verlag KG. 9677826

[115] Bernstein, E., Putnam, F. & Carlson, E. Development, Reliability, and Validity of a Dissociation Scale. *J. Nerv. & Mental Dis.* **174**, 727–735. 10.1097/00005053-198612000-00004 (1986). 10.1097/00005053-198612000-00004 3783140

[116] Wingenfeld, K. et al. The German version of the Childhood Trauma Questionnaire (CTQ): preliminary psychometric properties. *Psychother. Psychosom. medizinische Psychol.* **60**, 442–450. 10.1055/s-0030-1247564 (2010). 10.1055/s-0030-1247564 20200804

[117] Krüger-Gottschalk, A. et al. The German version of the Posttraumatic Stress Disorder Checklist for DSM-5 (PCL-5): psychometric properties and diagnostic utility. *BMC Psychiatry* **17**, 379. 10.1186/s12888-017-1541-6 (2017). 29183285 10.1186/s12888-017-1541-6 PMC5704375

[118] Geisheim, C. et al. Das Brief Symptom Inventory (BSI) als Instrument zur Qualitätssicherung in der Psychotherapie. *Diagnostica* **48**, 28–36. 10.1026/0012-1924.48.1.28 (2002).

[119] Cackowski, S. & Schmahl, C. Research Training Group (RTG) / Graduiertenkolleg (GRK) 2350: “Impact of Adverse Childhood Experiences on Psychosocial and Somatic Conditions Across the Lifespan”. *Neuroforum* 25, 265–266, 10.1515/nf-2019-0022 (2019). Publisher: De Gruyter.

[120] Behzadi, Y., Restom, K., Liau, J. & Liu, T. T. A component based noise correction method (CompCor) for BOLD and perfusion based fMRI. *NeuroImage* **37**, 90–101. 10.1016/j.neuroimage.2007.04.042 (2007). 17560126 10.1016/j.neuroimage.2007.04.042 PMC2214855

[121] Whitfield-Gabrieli, S. & Nieto-Castanon, A. *Conn* : A Functional Connectivity Toolbox for Correlated and Anticorrelated Brain Networks. *Brain Connect.* **2**, 125–141. 10.1089/brain.2012.0073 (2012). 22642651 10.1089/brain.2012.0073

[122] Fan, L. et al. The Human Brainnetome Atlas: A New Brain Atlas Based on Connectional Architecture. *Cereb. Cortex* **26**, 3508–3526. 10.1093/cercor/bhw157 (2016). 27230218 10.1093/cercor/bhw157 PMC4961028

[123] Bijsterbosch, J., Smith, S. M. & Beckmann, C. *An Introduction to Resting State FMRI Functional Connectivity* Oxford University Press(Google-Books-ID, 2017).

[124] Hayes, A. F. *Introduction to mediation, moderation, and conditional process analysis: a regressionbased approach*. Methodology in the social sciences (The Guilford Press, New York ; London, 2022), third edition edn.

[125] Anderson, M. C., Bunce, J. C. & Barbas, H. Prefrontal-hippocampal pathways underlying inhibitory control over memory. *Neurobiology of Learning and Memory* **134**, 145–161. 10.1016/j.nlm.2015.11.008 (2016). 26642918 10.1016/j.nlm.2015.11.008 PMC5106245

[126] Ben-Shachar, M. S., Lüdecke, D. & Makowski, D. Effectsize: Estimation of effect size indices and standardized parameters. *Journal of Open Source Software* **5**(56), 2815. 10.21105/joss.02815 (2020).

